# Resistance of Polypropylene Membrane to Oil Fouling during Membrane Distillation

**DOI:** 10.3390/membranes11080552

**Published:** 2021-07-22

**Authors:** Marek Gryta

**Affiliations:** Faculty of Chemical Technology and Engineering, West Pomeranian University of Technology in Szczecin, ul. Pułaskiego 10, 70-322 Szczecin, Poland; marek.gryta@zut.edu.pl

**Keywords:** membrane distillation, oily wastewater, polypropylene membrane, membrane wettability

## Abstract

The influence of oil emulsion presence in the water on the course of water desalination by membrane distillation was studied. The feed water was contaminated by oil collected from the bilge water. The impact of feed composition on the wetting resistance of hydrophobic polypropylene membranes was evaluated during long-term studies. Two types of the capillary membranes fabricated by thermally induced phase separation method were tested. It has been found that these membranes were non-wetted during the separation of NaCl solutions over a period of 500 h of modules exploitation. The addition of oil (5–100 mg/L) to the feed caused a progressive decline of the permeate flux up to 30%; however, the applied hydrophobic membranes retained their non-wettability for the consecutive 2400 h of the process operation. It was indicated that several compounds containing the carbonyl group were formed on the membranes surface during the process. These hydrophilic compounds facilitated the water adsorption on the surface of polypropylene which restricted the oil deposition on the membranes used.

## 1. Introduction

In recent years, significant progress in the development of membrane distillation (MD) has been made. It has allowed the application of MD to pilot plants with various manufactured constructions produced on an industrial scale [1,2,3,4,5,6,7]. The pilot plants are frequently supplied with seawater or surface water. The water composition significantly affects the performance of MD installation, especially when the feed contains oils and low-surface tension contaminants [7,8]. Oil contamination in seawater may appear locally as a result of the discharge of bilge water from ships [9].

In the MD process, water evaporates across the pores of non-wetted membrane and is collected in the permeate stream, whereas non-volatile solutes are retained in the feed [1,2,3,4,5,6,7,8]. For this reason, the main direction of MD process development is water desalination [1,2,3,4,5,6,7,8,10,11]. A great advantage of MD is the fact that this process allows one to obtain fresh water also from brines, which cannot be realized using the reverse-osmosis process with regard to a high osmotic pressure [1,11,12,13].

The MD process is also proposed for the treatment of saline wastewaters, including water produced from hydraulic fracturing and oil extraction [7,8,14,15,16]. In each of these cases, the feed may contain dispersed oily pollution and different chemical compounds (e.g., surfactants), which stabilize oil-in-water emulsion [7,8,16,17]. It must be pointed out that the effectiveness of oil separation from such polluted water with the traditional method is limited and definitely better results can be achieved using the membrane processes, particularly ultrafiltration (UF) [18,19,20]. However, the UF process cannot be used for water desalination, which is of particular concern when a zero-liquid-discharge technology is implemented [7,18,21,22]. Brines can be separated by an MD process, but the presence of oils and surfactants poses a serious problem associated with membrane durability (non-wettability) [8,17,23,24]. Generally, in an MD process the hydrophobic microporous membranes are applied, and with regard to this, an adsorption of petroleum derivative compounds (oils), exhibiting the hydrophobic properties, on the hydrophobic membrane surface can take place [8,17,25]. As a result, the hydrophobic MD membranes are wetted quickly, and the implementation of the MD process would not be possible [23,24,25].

In order to enable the treatment of oily wastewater by MD process, new types of membranes have been developed. Importantly, these membranes besides non-wetting by water are also resistant to wetting by oils, grease, and surfactants. Such properties were obtained by covering a thin hydrophilic layer on the surface of conventional hydrophobic membranes made from, e.g., polytetrafluoroethylene (PTFE) or polyvinylidene fluoride (PVDF) [17,26,27]. Although the presented composite membranes are significantly more resistant to fouling by oils, their main drawback is a reduction of their permeability resulting from the presence of additional layers [26,27,28]. Another option is membranes with the omniphobic properties of which the surface exhibits a hydrophobic and oleophobic nature and protects the membrane pores against the wetting by water as well as by low-surface tension contaminants present in the feed [8,29,30,31]. The hydrophobic membranes can also be protected via the aeration of modules. Surfactants and oil are adsorbed on the surface of air bubbles, which eliminates the interaction of these feed components with the membranes, and as a result, the wetting of hydrophobic membranes is significantly limited [32]. A good solution is also an integration of MD with other processes, which allows removing the oils and surfactants from water before it flows into the MD installation [7,15,18].

Oil fouling caused that the MD installation applied to desalinate water permanently contaminated by oils have different properties than the one designed for seawater desalination. In the first case, the results so far presented in the literature [8,15,16,17,18,23,24,25,29,30,31] unambiguously indicate that the feeding of MD installation with water containing petroleum derivative compounds requires pre-treatment stage as well as the application of membranes with enhanced resistance to wetting by these substances. Probably, such security/technological solutions do not have an MD installation applied for desalination of clean saline water using highly hydrophobic traditional MD membranes. In this case, the question arises as to whether the MD modules could be destroyed when accidentally, e.g., seawater contaminated by bilge water or other oily wastewaters generated by maritime transportation, flows into the MD installation.

Some authors have pointed out that in the case of the feed containing smaller amounts of oily derivative pollutants, a good resistance to the wetting can also be achieved using the traditional hydrophobic membranes made from polypropylene (PP) and PTFE [13,33]. This indicates that the feeding of MD installation with water containing a slight quantity of oils should not cause damage to MD modules. However, a significant effect of salt on organic fouling was also reported in other studies [34,35,36]. Therefore, it is difficult to unambiguously determine which effect causes the incorporation of even a slight oils amount into the MD installation desalting seawater, particularly when the salt concentration in the feed is significantly increased due to the application of a high recovery level of water. Moreover, the resistance of the membranes to wetting by oils was usually studied in the MD tests not exceeding 10–50 h. Such short time may make it difficult to assess this effect because the wetting process can take place very slowly. For this reason, in this work, the long-term MD studies were carried out using saline waters, which also contained small quantities of oil.

The capillary polypropylene membranes fabricated by thermally induced phase separation (TIPS) were applied in MD process [33,37,38]. The commercial Accurel PP membranes produced via TIPS method were not wetted in continuous MD research for over three years [39]. Hydrophilization of the surface of these membranes by gas plasma treatment allowed one to give PP membranes oleophobic properties [40]. As a result of the action of plasma gas, aldehydes, ketones, esters, and carboxylic acids were formed on the membranes surface, which led to local hydrophilic properties. The analysis of the surface composition of capillary PP membranes produced by various companies via the TIPS method showed that smaller amounts of such compounds containing carbonyl groups are also formed on the membranes surface [38,40,41]. Their presence should limit the adsorption of oil to polypropylene. For this reason, the aim of the research was to show whether the membranes produced by the TIPS method show good resistance to oil fouling and wetting during long-term separation of the feed containing small amounts of oil impurities.

## 2. Materials and Methods

### 2.1. MD Installation

The MD process was investigated in a variant of direct contact MD (DCMD) using two types of Accurel PP polypropylene capillary membranes (Membrana GmbH, Wupperta, Germany). These commercial membranes are manufactured for microfiltration (MF) using TIPS method. Although they were designed for the MF process, they exhibit good resistance to wetting by various solutions, which allowed them to be used for many applications of the MD process [33,39,40]. The membranes received from the manufacturer were used for MD tests without additional pretreatment.

The Accurel PP membranes were characterized by sponge structure with porosity of about 70%, and the average pore size amounted to 0.22 μm (manufacture’s specification). The internal/external diameters of the Accurel PP S6/2 and Accurel PP V8/2 HF membranes were equal to 1.8/2.6 and 5.5/8.5 mm, respectively. A submerged variant of the capillary modules was applied for MD tests. Three capillary membranes were mounted in S6 module, with an effective length of 25 cm, resulting in an outer surface area of 61 cm^2^. Only a single Accurel PP V8/2 membrane with length of 20 cm was assembled in the V8 module, which allowed one to obtain the module area equal to 53 cm^2^. Duplicated two modules of a given type (marked as A and B) were made for the MD tests.

**Figure 1:**
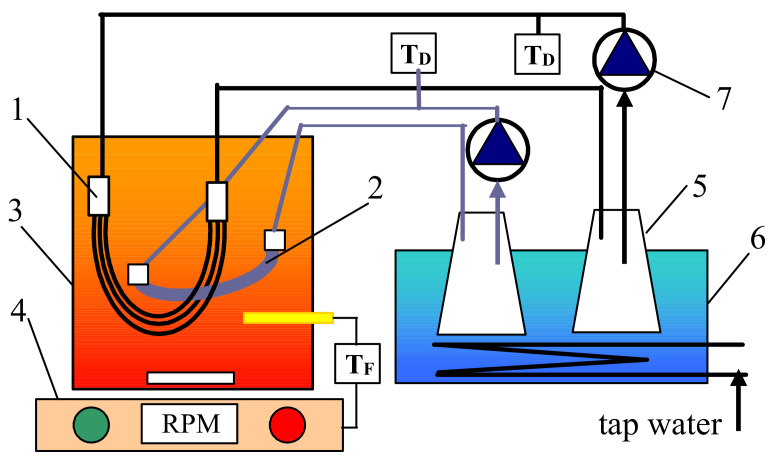
Scheme of the MD installation with submerged modules. 1—S6 module, 2—V8 module, 3—feed tank, 4—magnetic mixer with heating element, 5—distillate tank, 6—cooling bath, 7—peristaltic pump, T_F_—feed temperature, and T_D_—distillate temperature.

The tested MD modules were placed in a feed tank that was electrically heated and mixed (700 W, 500 rpm magnetic stirrer RCT Basic, IKA, Staufen, Germany). The distillate was circulated through a thermostatic loop (Figure 1). The flow rate of distillate amounted to 150 ± 5 mL/min, which allowed one to obtain the linear velocity of 0.32 m/s (S6) and 0.1 m/s (V8). The process temperature was equal to 291 ± 2 K for distillate and 323 ± 0.2 K for the feed. The initial volume of liquid in the glass tanks was 1.5 L (distillate) and 4 L of the feed.

For a given process parameters setting, the MD installation was operated continuously (day and night). The permeate flux [L/m^2^h] was calculated every 24 h, based on the volume increase of water flowing on the distillate side. The changes of process efficiency were calculated as a relation of the actual permeate flux to its initial values (E [%] = 100 J/J_0_).

### 2.2. Feed Solutions

The MD studies were performed using the solutions with salt concentration in the range of 1–100 g/L. Distilled water and pure NaCl (ChemPur, Karlsruhe, Germany) were used for their preparation. 

A volume of emulsion concentrate (dosed to the feed) necessary to obtain an assumed oil concentration in the feed was determined based on an analysis performed with an oil content analyzer (OCMA 500, Horiba, Kyoto, Japan). The small quantities of oily contaminants in the seawater originated mainly from bilge water discharged from ships [9]. For this reason, the oil used in the presented studies was collected from an engine room in a ship. This was heavy machine oil leaked from the piston seals of a marine engine. The oil emulsion concentrate was prepared by adding 5 mL of such oil to 1 L of deionized water. Subsequently, the content was intensively shaken for 5 min, and then the mixture was subjected to ultrasound treatment (620 W, Sonic-6D, POLSONIC, Warszawa, Poland) for 2–3 h. This operation was periodically repeated (every 2–3 days), which allowed one to stabilize the emulsion without adding surfactants. A fresh oil emulsion concentrate was prepared every two weeks.

### 2.3. Analytical Methods

The membranes resistance to wetting was tested by measurements of the electrical conductivity of water flowing on the distillate side as well as the concentration of oil and organic compounds (total organic carbon—TOC). Since the feed concentration was high, even a slight leakage of the feed through the wetted pores would cause significant changes in the values of tested parameters. The determination of TOC values was carried out using Multi N/C (Analytic Jena, Jena, Germany) analyzer, whereas the conductivity measurements were performed with apparatus 6P Ultrameter (Myron L Company, Carlsbad, CA, USA).

The oil content in the solutions was examined by infrared method using an oil analyzer OCMA 500 manufactured by Horiba (Kyoto, Japan). This apparatus performs an automatic extraction of oil from aqueous solutions with S316 solvent (Horiba, Kyoto, Japan). This analyzer was also applied for determination of the amount of oil adsorbed on the surface of the membranes. The membrane sample was washing by soaking into S316 solvent (20 mL), and obtained solution was appropriate diluted before the measurement. The obtained oil concentration was recalculated as mass of oil adsorbed on the membrane area [g/m^2^]. 

Mastersizer 3000E instrument (Malvern Instruments, Malvern, UK) was used for the determination of oil droplet size distribution. The obtained average values resulted from three measurements.

A Sigma 701 microbalance (KSV Instrument Ltd., Helsinki, Finland) was applied for measurements of the surface tension of solutions and the contact angle of the membranes. The studies were carried out using the Wilhelmy plate method at room temperature (293–294 K). 

The composition of the membrane surface was tested using the attenuated total reflection-Fourier transform infrared spectroscopy (ATR-FTIR). These analyses were performed using a Nicolet 380 FTIR spectrophotometer coupled with Smart Orbit diamond ATR accessory (Thermo Electron Corp., Waltham, MA, USA).

The surface morphology of the membranes was examined by atomic force microscopy (AFM). A multi-Mode 8 AFM apparatus equipped with a Nanoscope V converter from Bruker (Santa Barbara, CA, USA) characterized the membrane roughness in the scanasyst mode. The changes of membrane morphology were analyzed by scanning electron microscopy (SEM) observations (Hitachi SU8000, Tokyo, Japan). The SEM microscope was connected with energy-dispersive X-ray spectrometer (EDS) (Hitachi, Tokyo, Japan). 

## 3. Results and Discussion

### 3.1. Water Desalination

**Figure 2:**
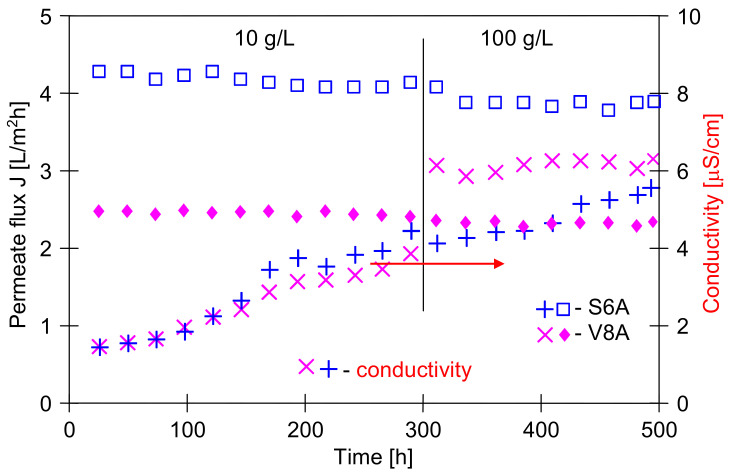
The changes of the permeate flux and distillate conductivity during MD process. Feed: NaCl solutions (10 and 100 g/L). Modules S6A and V8A.

Oil substances contaminating the feed can cause fouling and a partial wetting of the membranes [8,13]. The effect of such phenomena results in a systematic decrease in the efficiency of the MD process [17,33]. The quality of the distillate obtained are also deteriorated due to the permeation of feed constituents, and it is manifested by an increase in the electrical conductivity and content of organic compounds in the distillate. To obtain the reference parameters, the influence of MD process parameters on the membrane efficiency been investigated. For this purpose, the MD modules with the NaCl solutions as a feed (non-fouling solutions) were examined in the first stage of MD studies (Figure 2).

A salt dissolved in the water decreased the vapor pressure. For a saturated NaCl solution (5.5 M), the mole fraction is x = 0.09; hence, according to Raoult’s law (p = p^0^ − p^0^x), the driving force of the MD process decreases by 9%. In the studied case, an increase of the feed concentration to 100 g/L caused the permeate flux decline, from 4.3 to 3.9 L/m^2^h (S6A) and from 2.5 to 2.25 L/m^2^h (V8A), i.e., in both cases the flux decreased by 9%. This indicates that, due to the concentration polarization, the NaCl concentration at evaporation surface was significantly higher than in the feed (100 g/L, x = 1.71). However, despite such a significant increase of the salt concentration decreasing of the process, efficiency was insignificant. Such a low effect of salt concentration on the decrease of MD permeate flux is an important advantage of MD process [1,2,3,4,5,6,7]. 

The results presented in Figure 2 were obtained by conducting the MD studies for a period of 500 h. The electrical conductivity of water recycled on the distillate side did not exceed 7 μS/cm over this period, even during the separation of NaCl solution with the concentration of 100 g/L. This indicates that the modules construction was tight (particularly membrane potting in the module head) and the membrane used was characterized by the coefficient of salt retention equal to almost 100%.

**Figure 3:**
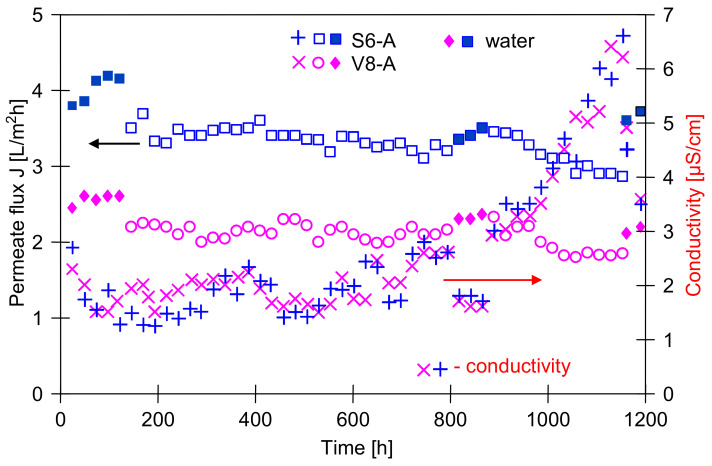
The changes of the permeate flux and distillate conductivity during MD process. Feed: water and NaCl solution (1 g/L) with oil (25 ± 5 mg/L).

In the next stage of studies, the NaCl solution was replaced by distilled water. The permeate flux obtained for both modules (S6A and V8A) after 500 h of MD module exploitation was close to the initial flux (Figure 3, water as a feed), which confirmed the previous observations that the used polypropylene membranes are resistant to wetting by NaCl solutions [37]. A similar resistance to the wetting by pure NaCl solutions was also observed for other hydrophobic membranes, such as PVDF [34]. 

### 3.2. MD Process of NaCl Solutions with Oil

It is well known that oil adsorption on the membrane surface can change a membrane performance [14,23,24,25]; thus, in the subsequent stage, the objective of the study was to determine the influence of the small amounts of oil in the feed on the course of MD process. The investigations were started from desalination of NaCl solution (1 g/L) that contained 25 mg/L of oil (Figure 3). It has been reported that the presence of oil in the feed caused a decrease of the permeate flux from 4.2 to 3.5 L/m^2^h (S6A) and from 2.7 to 2.2 L/m^2^h (V8A). The MD process was carried out for almost 1200 h, and a small but systematic decrease in the permeate flux value was observed. The largest decline was noticed after 900 h of MD. During the consecutive 300 h of studies, the permeate flux was reduced to 2.9 L/m^2^h (S6A) and to 1.92 L/m^2^h (V8A).

A reason for the noted decline of the MD modules efficiency could be associated with the membrane wetting resulting from the adsorption of oil [27]. However, on the distillate side only a slight increase of the water electrical conductivity was observed. In the final stage of the studies, the conductivity increased to 7 μS/cm (Figure 3). Considering that the feed comprises a solution containing about 1 g/L NaCl (conductivity over 2000 μS/cm), such low values of distillate conductivity indicate that only few pores in the membranes underwent the wetting. Since the module productivity obtained for distilled water also decreased (E = 12% for 6A, and E = 25% for V8A), it can be assumed that a layer of oil was formed on the membrane surface, which hindered the water transport to the evaporation surface. A similar result, i.e., a small decline of efficiency and a slight increase of distillate conductivity was obtained by other studies [34], who used the PVDF membranes for the separation of NaCl solutions (1 and 10 g/L) containing 100 ppm of oil. 

**Figure 4:**
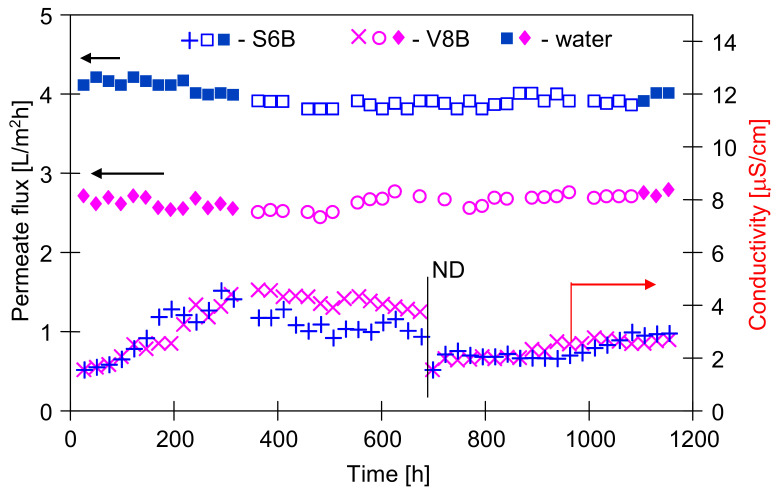
The changes of the permeate flux and distillate conductivity during MD process. Feed: water + 1 g salt/L and NaCl solution (10 g/L) with oil (50 ± 5 mg/L). ND-distilled side was re-filed with freshly distilled water. Modules S6B and V8B.

**Figure 5:**
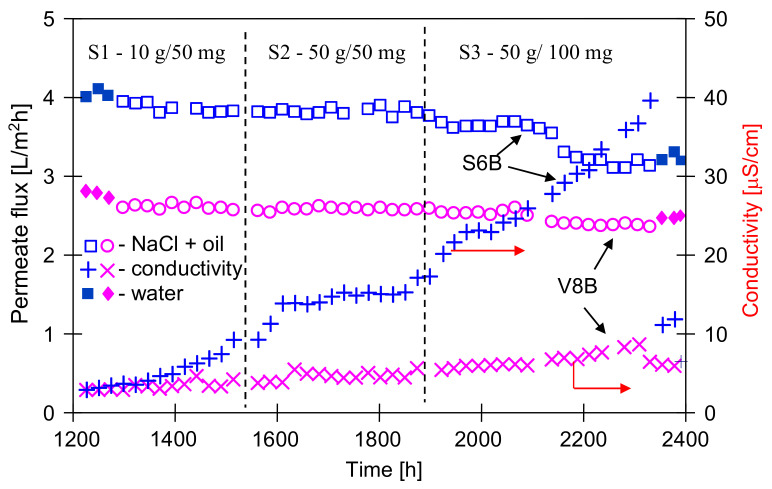
The changes of the permeate flux and distillate conductivity during MD process. Feed: S1—10 g NaCl/L + oil (50 ± 5 mg/L), S2—50 g NaCl/L + oil (50 ± 5 mg/L), and S3—50 g NaCl/L + oil (100 ± 15 mg/L).

The above positive MD results relate to low oil concentrations in the feed; however, it can be expected that a significant increase in oil content accelerates the wetting of membranes [27,28]. Therefore, in the next stage, the influence of oil fouling on the course of MD process was examined by increasing the oil concentration in the feed. The studies were carried out for the feed containing 50 mg/L (Figure 4) and 50–100 mg/L (Figure 5) of oil.

The experimental results demonstrated that an increase of the oil concentration in each case caused a slight decline of the permeate flux. The new MD modules (S6B and V8B) were applied in this part of studies. The maximal permeate flux was determined using water with 1 g NaCl/L as a feed (Figure 4, first 360 h). During the initial period of process, the distillate conductivity was increased to about 4 μS/cm. In the following hours, during the separation of the emulsions, this value did not increase, and even slightly decreased, although the salt concentration in the feed increased to 10 g/L. After about 800 h of MD process, the efficiency of the modules was close to the initial value. These results indicate that, in this case, the membranes have shown to be resistant to both oil adsorption and oil emulsion wetting. This is a slightly better result than that obtained for the membranes mounted in modules S6A and V8A (Figure 3), but these modules during the initial period (Figure 2) were fed with concentrated NaCl solution (100 g/L), which could change their surface properties.

The modules S6B and V8B were operated for a total of 2400 h, and a slight increase in the conductivity of the distillate was observed after 1300 h of the process (Figure 5). It is worth noting that in both cases (modules A and B) the increase in conductivity occurred after the modules were flushed with water (Figure 3, 850 h and Figure 5, 1250 h). A similar effect was observed in the case of scaling, where washing away the salt sediment from the membrane surface accelerated the wetting of the pores inside their walls [32,38].

After an initial increase of the distillate conductivity, its value was stabilized for oil concentration equal to 50 mg/L, although the salt concentration was enhanced from 10 to 50 g/L (Figure 5, from 1300 h). Increasing the oil content from 50 to 100 mg/L caused another increase in the conductivity of the distillate and a decrease in the permeate flux, which stabilized at the level of 3.3 L/m^2^h (S6B) and 2.35 L/m^2^h (V8B). This indicated that equilibrium in the thickness of fouling layer as a function of the feed concentration was obtained. The differences in the amount of adsorbed quantity of oil (as a function of oil content) were also found during the investigations of the emulsions (5–50 ppm of oil) separation through the PTFE membranes [42]. In the previous work [40], it was shown that at higher oil concentrations (100 mg/L) during the MD process, about 8 g/m^2^ of oil was adsorbed on the Accurel PP membranes surface.

The adsorption of oil on the membrane surface can accelerate the pores wetting. However, in spite of using a concentrated solution of NaCl (50 g/L), the final distillate conductivity 40 μS/cm was achieved for S6B module and 10 μS/cm for V8B module (Figure 5). Moreover, the oil concentration in the distillate was close to OCMA 500 analytic zero (0.1–0.2 mg/L), and determined TOC values were equal to 1.2 and 1.1 mg/L for S6B and V8B module, respectively. These results indicated that the used PP membranes demonstrated a good resistance to wetting by the feed containing both NaCl and oil (up to 100 mg/L). 

As expected, the Accurel PP V8/2 HF membrane with the fourfold wall thickness showed a greater resistance to wetting. However, a larger difference between this membrane and the Accurel PP S6/2 membrane was found for the operation time above 2000 h of MD process, because after 1200 h of the module operation, the distillate conductivity for both types of membranes was similar (Figure 3 and Figure 5). This result confirms that in order to demonstrate the reliable influence of the feed composition on the wetting of a given membrane, the MD process should be carried out for at least several hundred hours.

### 3.3. Membrane Wetting and Fouling

**Figure 6:**
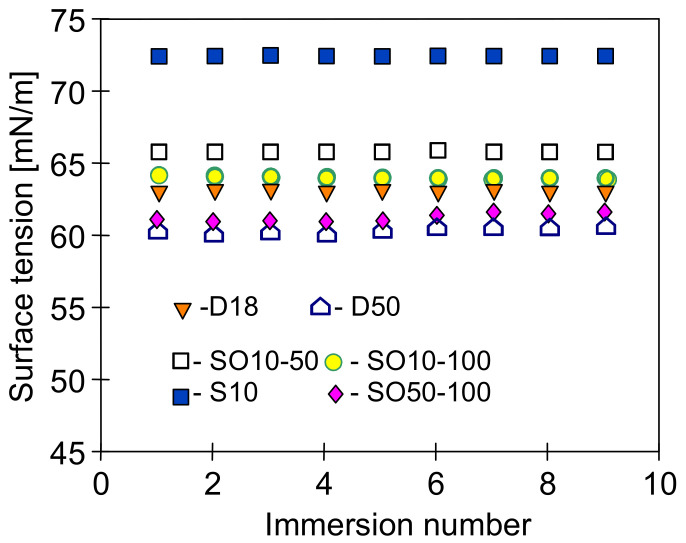
Measurement results of surface tension using Wilhelmy plate method. D—distilled water with oil (18 and 50 mg/L), S10—NaCl solution 10 g/L, and SO—NaCl solution (10 and 50 g/L) with oil (50 and 100 mg/L).

The feed surface tension (γ_F_) is one of the main parameters affecting the performance of MD process [43]. In the present study, it was determined that the surface tension for NaCl solution (10 g/L) was equal to 72.84 mN/m (Figure 6) and was a slightly higher than the value determined for deionized water. However, the presence of contaminants (oils) considered in this work can significantly reduce the surface tension of the feed and, as a result, may cause wetting of the membranes. Considering a value of the free surface energy of polypropylene determined in the range of 28–35 mJ/m^2^ [44,45], the values of γ_F_ should be above 30 mN/m [23]. 

In the presented research, the applied NaCl solutions containing small amounts of oil maintained a high surface tension value, similarly to the emulsions based on distilled water (Figure 6). The largest decrease in the surface tension was recorded for distilled water, for which the surface tension decreases with increasing oil concentration. The NaCl solutions have a more complex composition, which affects the membrane interactions with pollutants such as oils and surfactants [12,18]. Moreover, both the ions concentration as well as their type may affect the surface tension of the feed containing organic compounds [42,46]. However, in each of the studied cases, after adding the oil emulsion to the feed, its surface tension was significantly higher than the critical value for PP membrane (30 mN/m).

**Figure 7:**
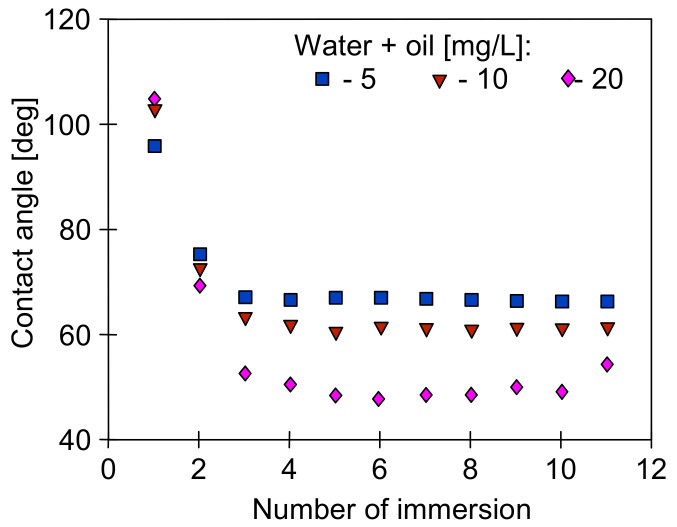
The changes of the advancing contact angle during continuous measurement of dynamic contact angle using Wilhelmy plate method. Samples of new (dry) Accurel PP S6/2 membrane immersed in different emulsions.

It is well known that the surface tension of the feed affects the contact angle of the membranes. It is assumed that membranes in the MD process are not wetted if the contact angle is at least 90 degrees [8,17,25,26]. Apart from the feed properties, this value is also dependent on the type of membranes used [8]. The contact angle values obtained for the PP membranes used in the presented work are shown in Figure 7. 

The composition of the tested solutions significantly affected the value of the contact angle, which was stabilized in the range of 50–75 deg (Figure 7), i.e., below the required 90 degrees. However, the contact angle value obtained after the first immersion of the membrane should be distinguished in the Wilhelmy plate method used. Usually, this value is close to the contact angle value determined by the drop method in goniometers. In the studied case, these values were larger than 90 degrees (Figure 7, immersion number 1). Indeed, the measurements are disturbed when the feed contains the components that adsorb on the membrane surface. In such cases, there is a change in the mass of the membrane sample and the wettability of its surface after each immersion, which in turn leads to a change in the calculated value of the contact angle. Based on this phenomenon, membrane fouling research can be carried out using the Wilhelmy plate method [47].

**Figure 8:**
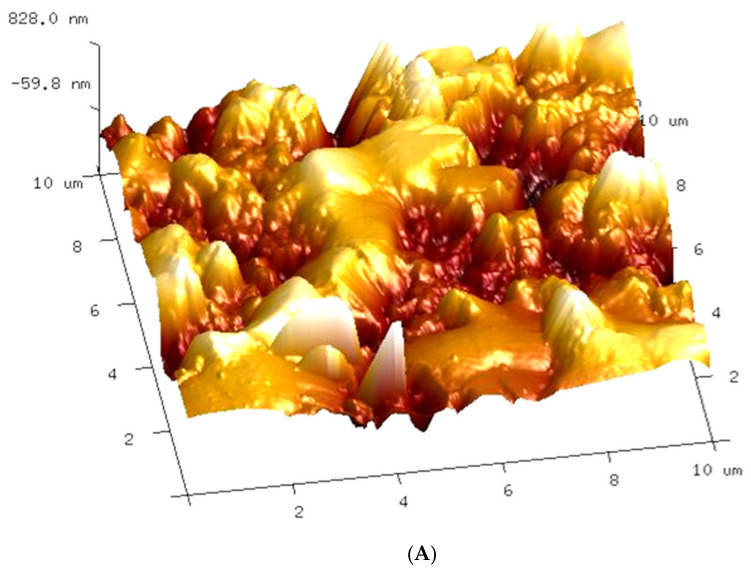

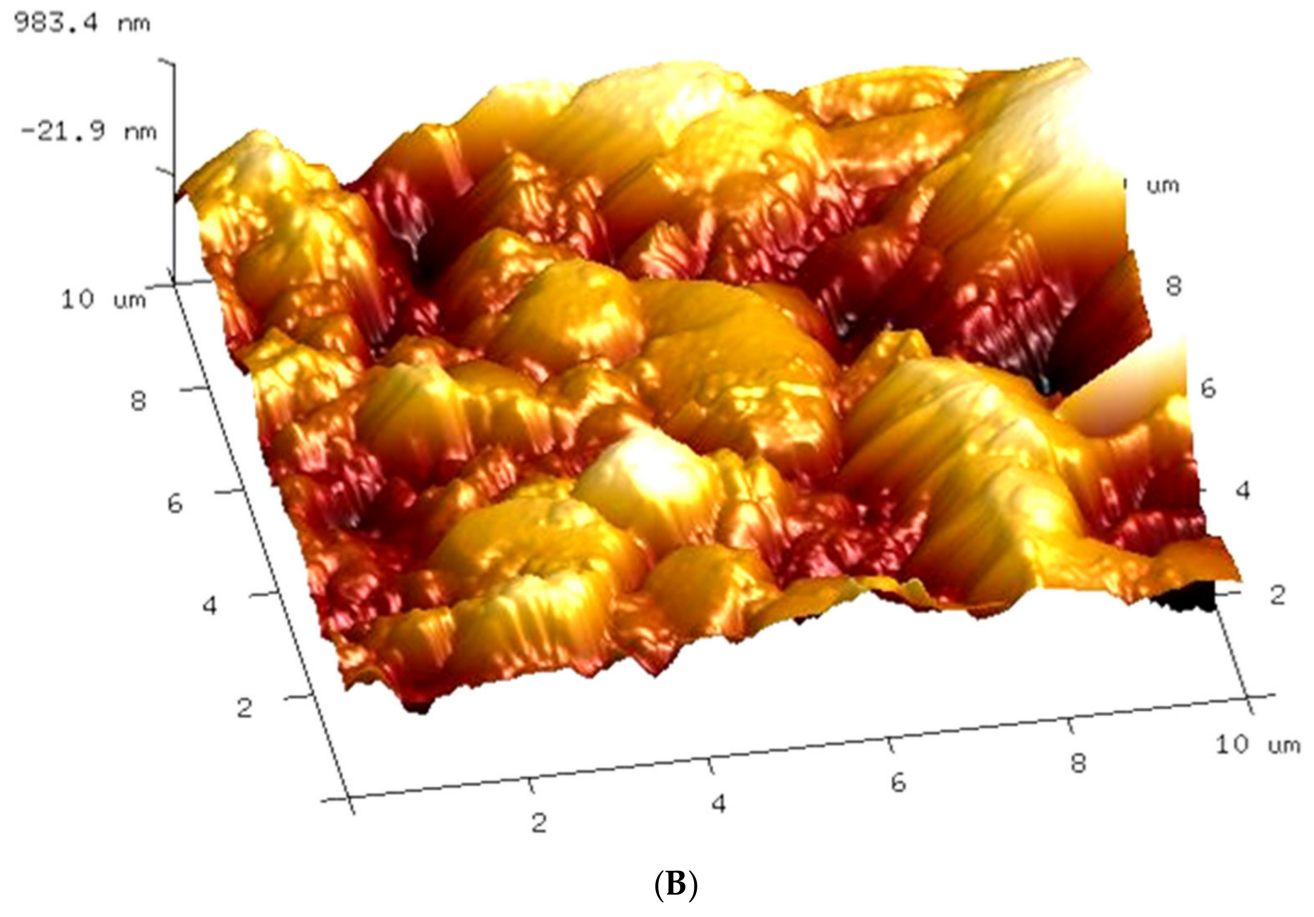
AFM image of Accurel PP V8/2 membrane external surface. (**A**) Virgin V8 membrane, (**B**) membrane collected from V8B module.

During measuring the surface tension of the tested emulsions (Figure 6), relatively stable values were obtained despite repeated Wilhelm’s plate immersion. However, the surface of this plate is highly hydrophilic, which limits oil adsorption. On the contrary, oil adsorption can be expected by immersing the hydrophobic membrane in the emulsion; hence, large changes in the contact angle were obtained (Figure 7) for the PP membrane (hydrophobic). It is worth noting that the intensity of such adsorption also depends on the membrane roughness and oil droplet size [8]. The AFM analysis showed a significant surface roughness of the used membranes (Figure 8), and the value of the average surface roughness (R_A_) was equal to 194 ± 34 nm (S6) and 206 ± 23 nm (V8). These mean values were calculated as the arithmetic mean of the R_A_ values obtained in the AFM tests for the five sites of the given membrane sample. After completion of the MD tests presented in Figure 5, the R_A_ values changed and were, respectively, 240 ± 29 nm for the membranes from the S6B module and 246 ± 54 nm for the V8B module.

**Figure 9:**
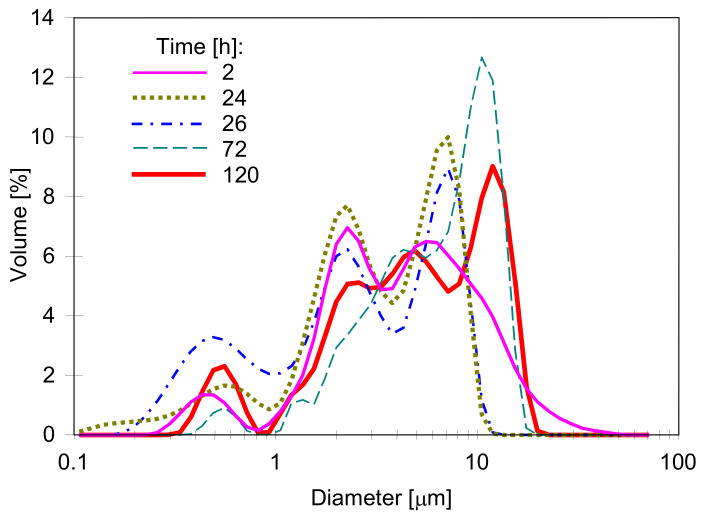
Changes in the oil droplet size distribution in the feed during the consecutive hours of MD test.

The study of the droplet size distribution showed that in the emulsions used, the size of the oil droplets was below 100 µm, with the dominant fraction in the range of 1–10 µm (Figure 9). Mixing the feed, oil adsorption on the membranes surface, and the feed tank as well as coalescence caused a systematic drop in the oil content, which was prevented by periodically adding successive portions of the emulsion concentrate. All these phenomena also caused changes in the droplet size distribution, but as shown in Figure 9, these changes were not too large.

**Figure 10:**
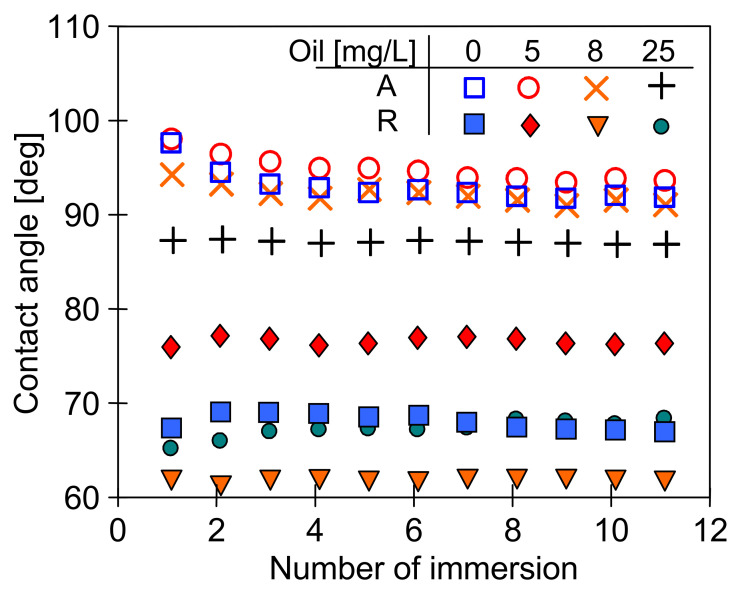
The changes of the advancing (A) and receiving (R) contact angle during continuous measurement of dynamic contact angle using Wilhelmy plate method. Sample—PP foil.

To reduce the effect of porosity and roughness on the oil adsorption intensity, additional measurements for a smooth PP film for comparison purposes were performed (Figure 10). In this case, much higher values of the contact angle (advancing) were obtained than those presented in Figure 7. Moreover, the angle values (receiving) obtained during the emerging of the foil samples were also much higher than those obtained during the measurements using the samples of PP membrane. The results (Figure 10) indicate that polypropylene is not easily wetted by the used solutions. It has been shown that the PP membranes exhibit a good wetting resistance, even when their surface was quickly wetted [48].

The above results (Figure 7) indicate that feeding the MD modules with the feed containing oil causes the oil droplet adsorption on the surface of the PP membranes tested. Membrane fouling caused by oil can lead to wetting of the membranes [8,17,23,24], which was not observed during the long-term MD studies (Figure 2, Figure 3, Figure 4 and Figure 5). It is known from Reference [48] that the tested Accurel PP membranes are wetted on the surface after about 50 h of the process which most probably limited the intensity of oil fouling. This was also confirmed by the results of a simple test. The 100 cm long Accurel PP S6/2 membrane was cut in half, and one part (for surface wetting) was placed in distilled water for 70 h. Both membranes were then immersed for a period of 2 h in an emulsion containing 102 mg/L of oil with the dominant fraction in the range of 1–10 µm. It was determined that after washing in solvent S316 (OCMA 500 analysis) 0.356 g/m^2^ of oil had adsorbed on the surface of the dry PP membrane, while on the surface wetted membrane, this amount was significantly lower (0.0971 g/m^2^).

**Figure 11:**
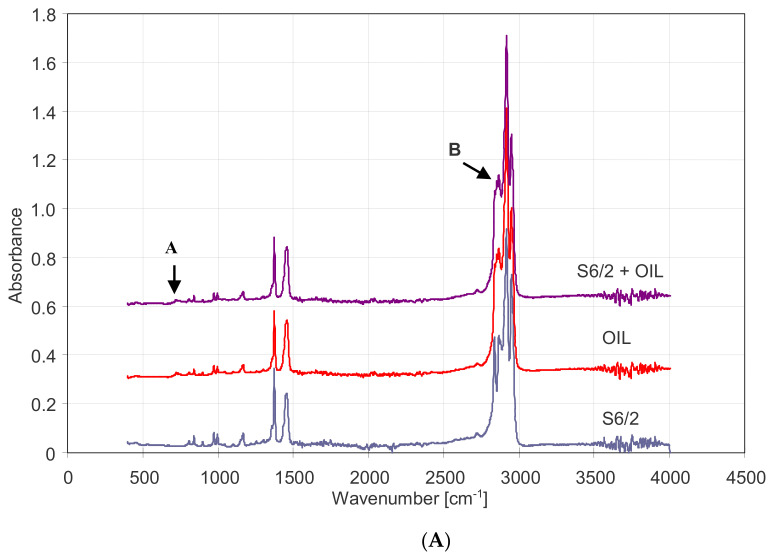

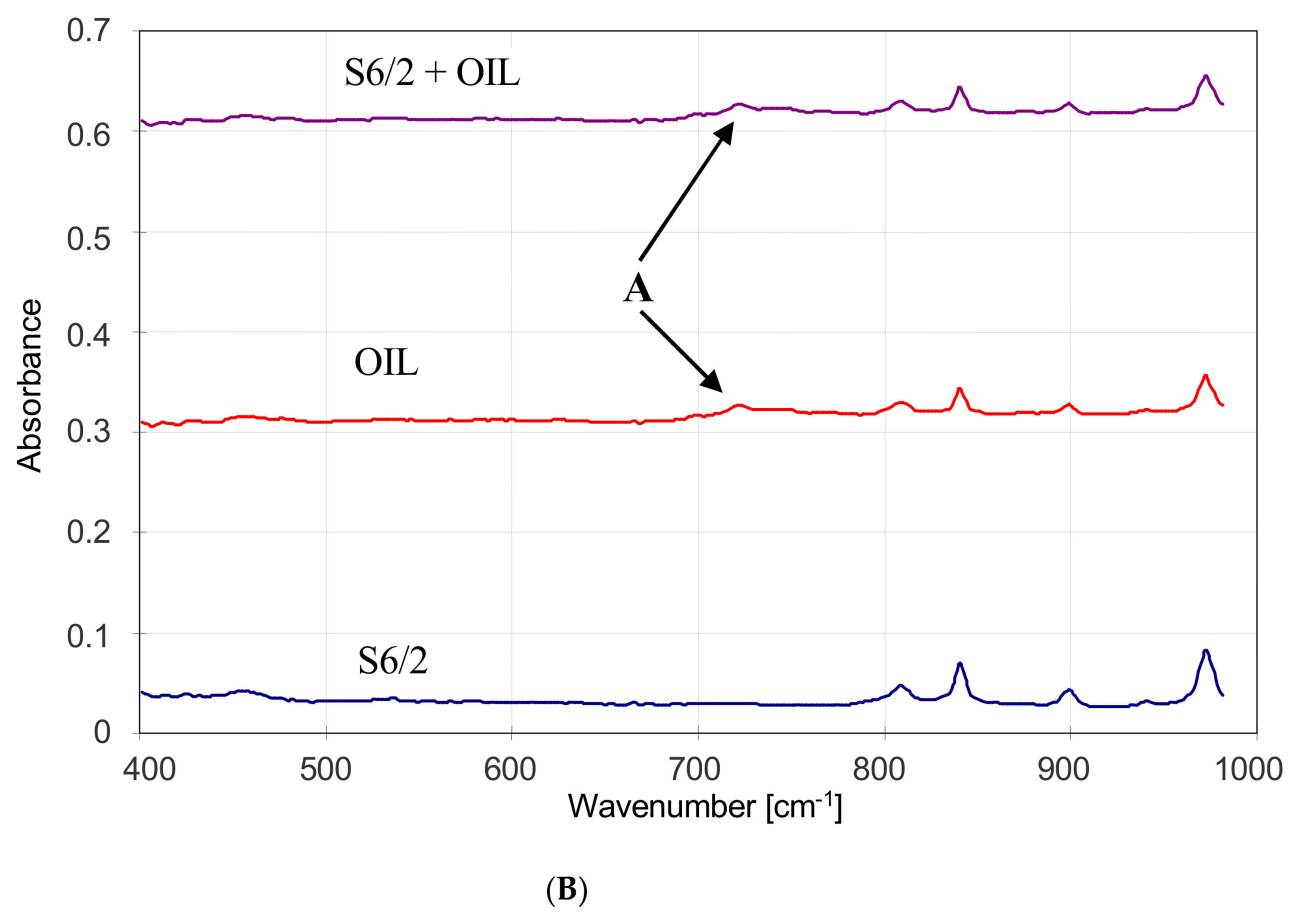
Comparison of FTIR spectra of the oil, virgin S6/2 membrane, and this membrane wetted by oil (**A**,**B**) zoom in of the region below 1000 cm^−1^.

**Figure 12:**
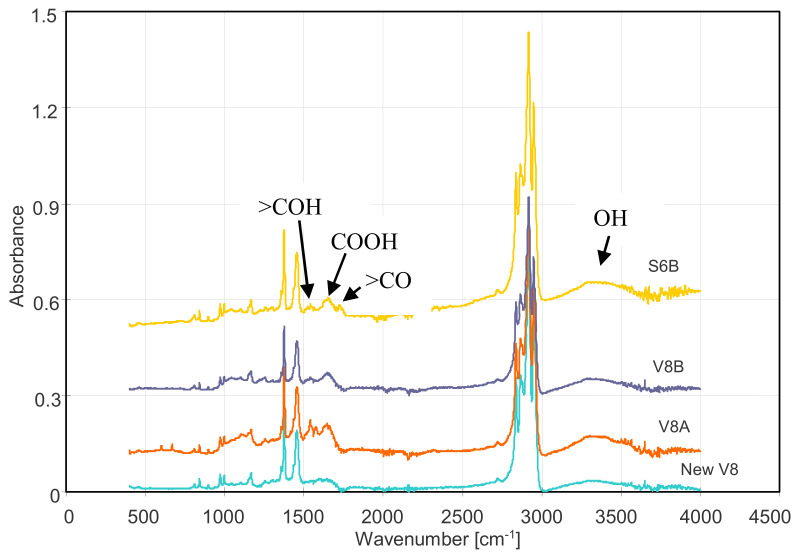
Comparison of FTIR spectra of the virgin V8 membrane and membranes collected from MD modules (V8A, V8B, and S6B) after MD tests.

Polypropylene is not wetted so quickly; however, as shown [40,41], the membranes produced by the TIPS method also have the hydrophilic groups on the PP surface, which facilitates the water adsorption on their surface. The number of hydrophilic groups on the surface of Accurel PP membranes increases significantly during the following hours of the MD process [38]. As a result, the membranes whose surface had hydrophobic–hydrophilic properties were obtained. This was also confirmed by FTIR studies of the tested membranes, which showed the presence of hydrophilic groups on the PP surface (Figure 11 and Figure 12). Similarly, anti-oil-fouling properties are obtained by covering the hydrophobic membranes with additional thin hydrophilic layers (Janus membrane) [17,26,27]. However, such additional modifications complicate and increase the production costs; hence, it is more advantageous to use the propensity of PP to an auto-oxidation processes [38,40]. As a result, the hydrophilic surface properties of membranes formed via TIPS can be intensified by pre-treating the membranes, starting the MD installation with a feed free from organic pollutants. 

It should be emphasized that the PP membranes were characterized by the increased resistance to oil fouling only after prolonged soaking in water, because a droplet of oil immediately penetrates the pores of a new and dry Accurel PP membrane. The results of the FTIR analysis for both wetted and non-wetted by oil membranes are shown in Figure 11. The obtained spectra have a characteristic for PP absorption bands corresponding to the vibration of the groups –CH, –CH_2_, and –CH_3_, e.g., methyl absorption band at 1375 and 1450 cm^−1^ [49] and C-H stretching (2837, 2866, 2917, 2949, and 2970 cm^−1^) [50]. 

The above-mentioned groups are also the basis for the composition of the oil; thus, the spectra obtained for an oil-wetted membrane are similar to spectra obtained for clean PP membranes (Figure 11). The slight differences are the small peak at 715–750 cm^−1^ (Figure 11B, point A) and the splice of the peaks in the range 2850–2890 cm^−1^ (Figure 11A, point B).

The FTIR spectra of the membranes after the MD process showed the appearance of new peaks in the range of 1500–1800 cm^−1^ (Figure 12), which were not obtained for the tested oil. In this range, the carbonyl groups presented in aldehydes, ketones, esters, and carboxylic acids have a strong absorption [51,52]. There was also an increase in the broadband in the 3000–3600 cm^−1^ range characteristic of the hydroxyl groups. These peaks were not observed during FTIR analysis of the interior of the membrane wall. After removal of the outer wall layer (0.2–0.3 mm), the obtained ATR-FTIR analysis results were similar to those obtained for the new membrane (Figure 12, new V8). This result indicates that the hydrophilic groups were mainly formed on the surface of the membranes.

**Figure 13:**
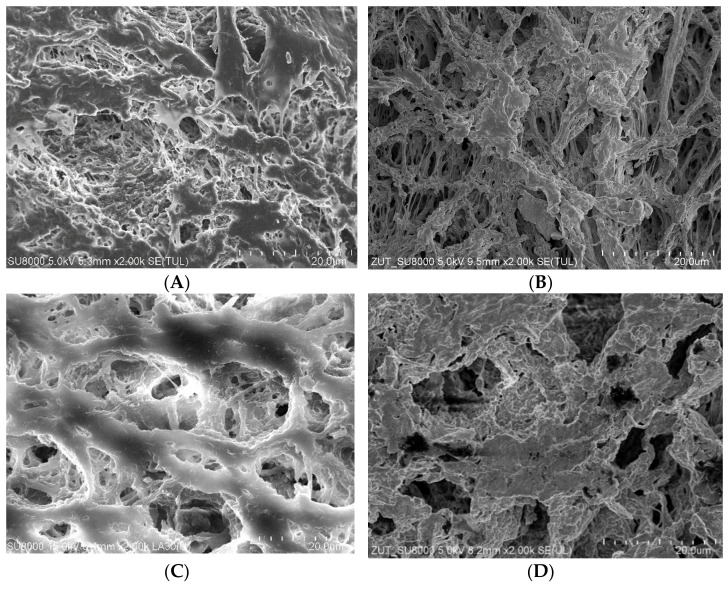
SEM images (magnification 2000×) of virgin Accurel PP membranes surface and these membranes with deposit formed during MD process of NaCl solution contaminated by oil. (**A**) Accurel PP V8/2, (**B**) module V8B, (**C**) Accurel PP S6/2, and (**D**) module S6B.

The SEM observations of the membrane samples showed that a small amount of sediment was formed on the surface of the tested membranes. The comparison of virgin membrane surfaces with sediment contaminated membranes is shown in Figure 13. These deposits did not form a solid layer and only locally covered the inlets to the pores. Their presence limited the flow of water to the membrane and was one of the main reasons for the observed slight decrease in the permeate flux during the MD process (Figure 3 and Figure 5). Moreover, the SEM observations of membrane cross-sections showed no sediment inside the walls, which confirmed that the tested oil-in-water emulsions did not penetrate the pores. SEM-EDS analysis showed the presence of a small amount of Na and Cl and trace amounts of S, Si, Mg, and K.

## 4. Conclusions

The long-term MD studies conducted for several thousand hours confirmed the good resistance of PP membranes to wetting during water desalination. However, unlike in the case of NaCl solutions, the separation efficiency of the MD process did not decrease during the desalination of feed water containing up to 100 mg/L of oil. This indicates that a slight amount of oil impurities, e.g., caused by bilge water, should not affect the seawater desalination by MD process.

Moreover, it has been demonstrated that increasing the wall thickness of the membrane reduces the permeate flux but significantly increases the wetting resistance of PP membranes.

The results of the MD studies indicate that it is advantageous if the hydrophobic MD membranes also contain small amounts of hydrophilic groups on their surface. In the case under study, the hydrophilic-compounds-containing carbonyl group were formed (as a side effect) on the membranes surface during the membranes production via the TIPS method, which gives the surface oleophobic properties of PP membranes. The number of hydrophilic groups increases during the MD process. Therefore, it is advantageous if the new MD modules are supplied with clean water (without the oil) during the initial 2–3 days, which allows them to obtain a membranes surface with hydrophobic–hydrophilic properties. Such membrane surface properties limit the intensity of the oil fouling, and as shown in the performed MD studies, a short-term contamination of the water feeding the MD installation by small amounts of oil should not cause wetting of polypropylene membranes.

## Data Availability

The data presented in this study are available on request from the corresponding author. The data are not publicly available due to the institutional repository being under construction.
